# An IoT Platform with Monitoring Robot Applying CNN-Based Context-Aware Learning

**DOI:** 10.3390/s19112525

**Published:** 2019-06-02

**Authors:** Moonsun Shin, Woojin Paik, Byungcheol Kim, Seonmin Hwang

**Affiliations:** 1 Department of Software, Konkuk University, Chungju 27478, Korea; msshin@kku.ac.kr (M.S.); wjpaik@kku.ac.kr (W.P.); 2 Department of Information and Communication, Baekseok University, Cheonan 31065, Korea; bckim@bu.ac.kr

**Keywords:** IoT platform, intelligent monitoring robot, active CCTV, learning model, machine learning, convolutional neural network

## Abstract

Internet of Things (IoT) technology has been attracted lots of interests over the recent years, due to its applicability across the various domains. In particular, an IoT-based robot with artificial intelligence may be utilized in various fields of surveillance. In this paper, we propose an IoT platform with an intelligent surveillance robot using machine learning in order to overcome the limitations of the existing closed-circuit television (CCTV) which is installed fixed type. The IoT platform with a surveillance robot provides the smart monitoring as a role of active CCTV. The intelligent surveillance robot, which has been built with its own IoT server, and can carry out line tracing and acquire contextual information through the sensors to detect abnormal status in an environment. In addition, photos taken by its camera can be compared with stored images of normal state. If an abnormal status is detected, the manager receives an alarm via a smart phone. For user convenience, the client is provided with an app to control the robot remotely. In the case of image context processing it is useful to apply convolutional neural network (CNN)-based machine learning (ML), which is introduced for the precise detection and recognition of images or patterns, and from which can be expected a high performance of recognition. We designed the CNN model to support contextually-aware services of the IoT platform and to perform experiments for learning accuracy of the designed CNN model using dataset of images acquired from the robot. Experimental results showed that the accuracy of learning is over 0.98, which means that we achieved enhanced learning in image context recognition. The contribution of this paper is not only to implement an IoT platform with active CCTV robot but also to construct a CNN model for image-and-context-aware learning and intelligence enhancement of the proposed IoT platform. The proposed IoT platform, with an intelligent surveillance robot using machine learning, can be used to detect abnormal status in various industrial fields such as factory, smart farms, logistics warehouses, and public places.

## 1. Introduction

The development of Internet of Things (IoT) technology makes it possible to connect smart objects together through the Internet [1]. Advancements in IoT technologies provide enormous potential for high-quality, more convenient, and intelligent service. Various researches on intelligent IoT service systems are attracting attention due to the development of IoT technology. Recent research shows more potential applications of IoT in information intensive industrial sectors. Various needs, such as automatic setting, autonomous control, and optimal operation, are emerging in addition to inter-object connectivity support in the IoT service system. Although it provides connectivity through internet and automation functions by presetting, it is difficult to maintain stable operation and continuous value creation in the application domain. User monitoring and intervention is needed to resolve these problems. The development and popularization of machine learning and deep learning technologies enable a variety of intelligent services and challenges that previously could not be solved. The intelligent IoT service system is defined as a system that acquires data from the environment, recognizes the situation using the acquired data, and interacts with the user environment according to the service rules and the domain knowledge [2]. Therefore, the accuracy of the context-aware learning model-based on the domain knowledge can influence the quality of the intelligent monitoring service. Many researches on intelligent-robot services regarding the various applications have been in progress [3]. Researches on intelligent robots are able to be applied to service and application based on specific domains such as education, entertainment, life, and manufacturing. We tried to combine intelligent robot service and IoT technology in order to create a new context-aware service.

In this paper we propose an IoT platform with an intelligent monitoring robot which monitors the surrounding environment to figure out the situation and inform the administrator when an abnormal situation occurs. Unlike the existing robot system with a separate server, the intelligent monitoring robot in the proposed IoT platform has not only a server built into it, but also many kinds of devices such as the webcam, the radio -frequency (RF), the ultrasonic sensor, temperature sensor, light sensor, and sound sensor. It is designed to provide convenient use of monitor and control at anytime and anywhere using Wi-Fi network.

Especially, the proposed system performs context-aware learning by using a convolutional neural network (CNN)-based machine learning for context-aware learning. CNN is a method of machine learning optimized for image learning because it can input two-dimensional structure. CNN has proven its superior performance in extracting high-level abstracted features from images and recognizing objects in an optimal way.

This paper is organized as follows. Section 2 briefly describes the related works and Section 3 presents the framework of an IoT platform with a monitoring robot. We designed the CNN model for context-aware service as outlined in in Section 4 and analyzed the experimental results as seen in Section 5. Finally, we describe the conclusions in Section 6.

## 2. Related Works

Many efforts have been conducted toward employing IoT technology in the various industrial field to acquire data, process data timely, and distribute data wirelessly [1]. In recent years, the CCTV video-surveillance system has been introduced into various industrial fields and it has developed into a network-based CCTV or an intelligent-CCTV. The intelligent-CCTV system has been evaluated for its ability to monitor situations very effectively as it can detect the characteristics of an object or a person automatically. Nevertheless, most CCTV systems have been installed in fixed positions, and send images to a central server [4]. Therefore, immediate response to risks and anomalies is difficult because monitoring can only be performed in the control center after the images have been sent to a remote server. It is possible for active CCTV with IoT to perform real-time, context-aware, and immediate response. Recently, the need for context awareness to serve as an intelligent service in ubiquitous environments has increased with the development of a variety of sensor technologies [5]. Many kinds of techniques, such as machine learning, Bayesian network, data mining, and collaborative filtering, are applied for the construction of context-aware models to provide customized intelligence services in a variety of domains [5].

Although various attempts have been made to provide a context-aware service, most of them are being developed as a monitoring framework [3]. In [6], they applied the CNN-scheme-based optical camera communication system for intelligent Internet of vehicles. They used CNN for precise detection and recognition of light-emitting diode patterns at long distances and in bad weather conditions [6]. It can be used to employ CNN in various ways. We propose to employ CNN in the proposed IoT platform with an intelligent surveillance robot and it can be utilized in various fields of surveillance.

A context-aware service can recognize the circumstances, and then provide an appropriate service according to the environment [7]. Recently, the need for a context-aware service as the intelligent service in ubiquitous environments according to the development of sensor technologies has increased [8]. Above all, a context-aware model must be constructed before the context-aware service can be applied. Various techniques such as machine learning, collaborative filtering, and Bayesian network can be used to build context-aware models to provide customized intelligence services in a variety of domains [9]. An ontology-based context model will be able to describe a context semantics method which is independent of system or middleware [9]. However, in the case of a context-aware learning model for image recognition or pattern recognition, there are limitations to an ontology-based context-aware model [10].

**Figure 1:**
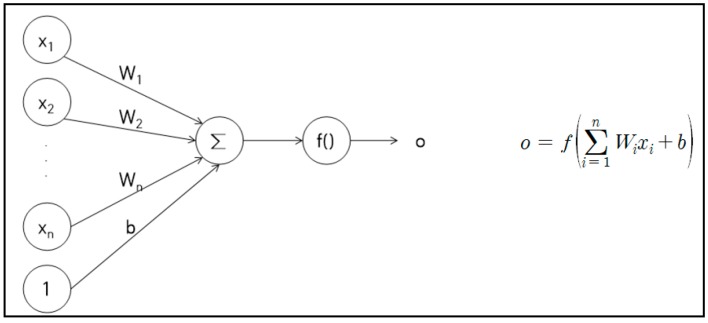
Neuron structure of artificial neuron network (ANN).

In order to overcome these limitations of an ontology-based context-aware model, we employed convolutional neural network (CNN)-based machine learning (ML), which was optimized for image- or pattern-recognition [11,12]. CNNs have developed significantly in recent years and are being used in a variety of areas, such as image and pattern recognition, natural-language processing, video analysis, and speech recognition. The improved network structures of CNNs lead to memory savings and reduced computational complexity and, at the same time, offer better performance for numerous applications. A CNN is composed of a series of layers, wherein each layer describes a specific function. The neuron structure of the artificial neural network is shown in Figure 1. In Figure 1, *x* is the input signal, *W* is the weight, *b* is the bias, *f* is the activation function, and o is the output. The active function f can be used as a sigmoid function, a hyperbolic tangent function, or a ReLU (rectified linear unit) function. In the case of image classification, the ReLU function has recently been used more than other functions because it shows better performance.

**Figure 2:**
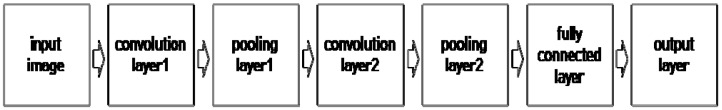
Architecture of convolutional neural network (CNN).

To find the optimal weight and bias during learning, the differential is used to get the slope, and the amount of change in the weight is an estimate of the slope. The learning rate is a value that determines how much the parameter value is updated in one learning cycle, and must be set when neural network modeling has been carried out. The learning rate could be a value between 0 and 1. The smaller the learning rate, the slower the speed of learning. If it is set large, the learning speed can be increased, but the neural network may become unstable. Because the neural network model learns the training data excessively in order to decrease the error rate, side effects like overfitting occur and cause it to increase the error rate for actual data. To avoid overfitting, a drop-out technique can be used, which does not use all the nodes of the neural network, but rather selects some of the nodes at random [13]. Whenever weights are updated, the nodes are randomly reconstructed, and learning is performed through them. In the case of using fixed constants as a learning rate, the learning may not be performed properly. To solve these problems Momentum, AdaGrad (adaptive gradient), and Adam (adaptive moments) algorithms have been proposed. Momentum algorithm adds a momentum term to the slope and applies the update more strongly when the slope is in the same direction as the momentum [14]. In our study we adopted ADAM to overcome the overfitting problem. When modeling CNN using a backpropagation algorithm, the performance depends on the initial value of the weight. Preliminary training can be performed using a restricted Boltzmann machine (RBM) or autoencoder to obtain the initial value of the appropriate weight. Hinton proposed the RBM which consists of one input layer and one hidden layer [14]. The initial value of the weight in autoencoder is obtained by preliminarily training performed in each layer of the neural network using the unsupervised learning algorithm. CNN (convolution neural network) is one of the deep learning methods and is used to analyze image data and classify it according to its features [14]. Each layer of CNN has a function o for extracting and learning features by applying a filter to the input image. The CNN is effective in learning on image recognition. The architecture of the CNN is shown in Figure 2.

As shown in Figure 2, CNN generates the output from the input image through the convolution layer and the fully connected layer. The convolution layer consists of several convolution layers and pooling layers. The convolution layer generates the convolution output using the input image and the filter and generates the feature map by applying the activation function to the convolution output. The pooling layer generates the output image by reducing the dimension of the feature map using the pooling function. The output image of the last pooling layer is used as the input of the fully connected layer. Yann LeCun developed LeNet5 in 1998 using CNN [15]. This technique has an effect on number recognition. It receives 32 × 32 image and generates output through three convolution layers, two pulling layers, and one fully connected layer. AlexNet, released by Krizhevsky, Sutskever, and Hinton at ILSVRC-2012, was awarded first prize with an error rate of 15.3%, which was remarkably excellent compared to second prize with a 26.2% of error rate. AlexNet consists of five convolution layers, one pooling layer, and three fully connected layers, using two GPUs [16]. ResNet has demonstrated that networks can be deepened to a maximum of 152 layers, and verified better results than fewer layers [17].

These previous works have found that as the depth of the layers becomes deeper, the accuracy of learning is improved in deep learning [18,19]. In our study, we extended LeNet-5 model for context-aware learning of IoT-based intelligent monitoring.

## 3. Framework of an IoT Platform with an Intelligent Monitoring Robot

**Figure 3:**
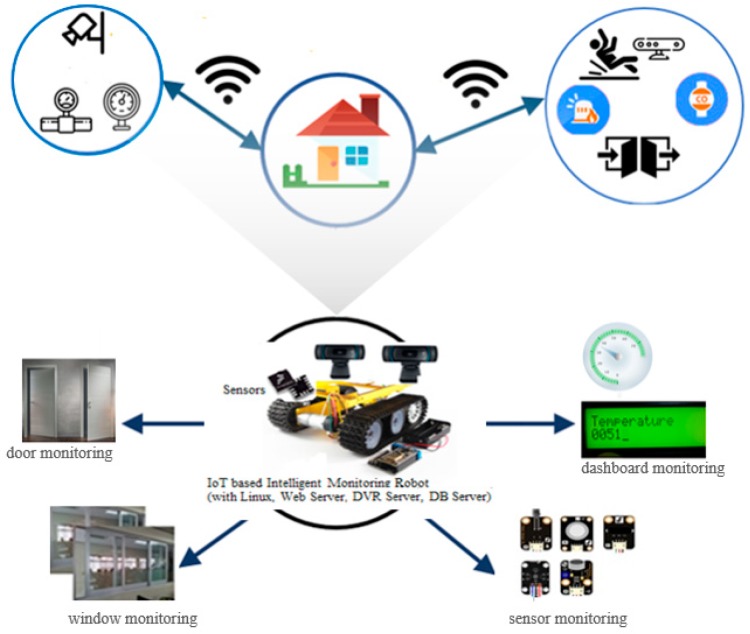
Framework of an IoT-based intelligent monitoring robot.

In this section, we present a system architecture of an IoT platform with an intelligent monitoring robot by applying a CNN-based context-aware learning model we designed. Figure 3 shows the framework of an IoT platform with an intelligent monitoring robot. As shown in the Figure 3, the IoT-based context-aware system must have its own server and communicate with the web or app client using Wi-Fi. The basic functions required for IoT-based intelligent monitoring systems are learning of situational awareness and real-time recognition process. In addition, in order to provide a notification service for monitoring results and for abnormality detection in real time, it is necessary to make real-time communication always possible by applying IoT technology. The IoT-based context-aware system can be implemented as a fixed or portable type. In the latter case, autonomous navigation using a line tracing or map of a specific area is required.

The proposed system needs to notify the situation through a smart phone and perform image processing functions for the detection of abnormal status. For this the system needs a web server, DB server, and DVR server to be constructed on the main controller in order to search the stored images at the remote site. These servers need to be capable of image storage, event storage, search, and then the web- or app-client will be able to control the movement of the robot for remote users and to receive images. The data transmitted from the attached sensor should be stored in the proposed IoT-based system and be displayed in real time on the remote web browser or app client. The image receiving function and the robot control function need to be supported for the app client, because it is necessary for the user to receive images and to control the movement of the robot by providing the management of a real time context-aware service.

**Figure 4:**
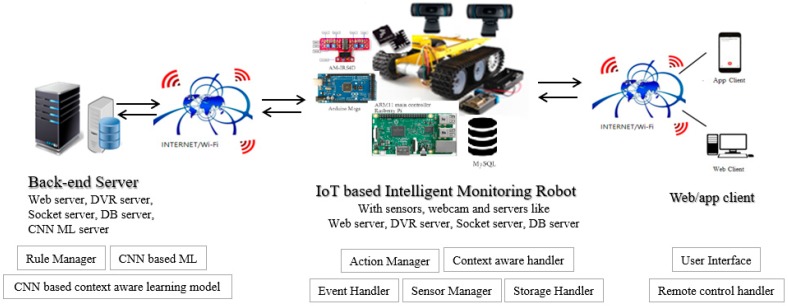
Architecture of the IoT platform with monitoring robot applying CNN machine learning (ML).

Figure 4 shows the architecture of IoT platform with an intelligent monitoring system that performs context-aware learning. An IoT platform with a context-awareness system should be able to distinguish between abnormal status and normal status from the monitored sensor value or images from a webcam by performing learning and real-time recognition processes on normal states to check for abnormal states. Also, it must have a variety of sensors that can measure indoor air conditions, such as room temperature and CO, and must be able to perform photo-taking functions to monitor the situation at a specific location. The sensors of the ultrasonic wave, the temperature, the light, the carbon monoxide, and the vibration will be used to detect the situation information of a predetermined area. It also should have a repository that stores the sensing values or images from a webcam while monitoring the environment. We constructed a web server, database server, socket server, and DVR server not only in the IoT-based monitoring robot but also in the backend server for backup and CNN-based context-aware learning.

Since the server itself is built in the body of the robot, it is able to check the streaming images and sensor value by using the remote client. Images are stored with date time, so it is possible to retrieve the image of the specific date and time by the client. The intelligent monitoring robot of the proposed IoT platform has a motor driver and servomotor, for moving the motor inside, and a infrared sensor for sensors and line tracing are connected to a separate battery and to an Arduino pin. Arduino is capable of serial communication with Raspberry Pie via USB port. Two webcams are used, which are streamed via the Mjpg-streamer. The one in the bottom is only used for streaming, and the other one attached to the servo motor can be used for streaming and image shooting. The hardware device could generate and send various sensor-values and image streaming, and then Node.js web server can parse the received sensor values and send them to the client. After being stored in the database, alarms can be generated abnormality is detected. The web or app client can request the client page via TCP/IP and retrieve the image of the desired date stored in the database. It can request streaming images from a webcam streaming server and receive the contextual information and sensor values in real time via sockets.

**Figure 5:**
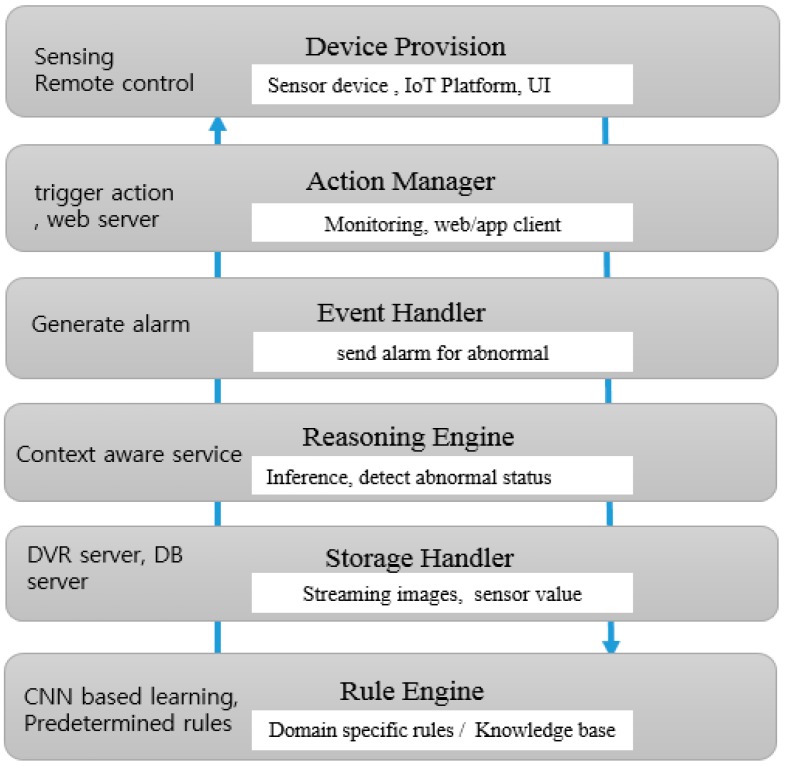
Architecture of the IoT platform with an intelligent monitoring system.

The software architecture of the IoT platform with an intelligent monitoring system is shown in Figure 5. It consists of five components: Action Manager, Event Handler, Storage Handler, Reasoning engine, and Rule Engine. Action Manager carried out actions like line tracing for monitoring.

Event Handler generates alarms when abnormal states are detected and Storage Handler stores the images from the webcams and values from sensors. Reasoning engine performs the functions to figure out whether there is an abnormality in situation information such as sensor values and images. Rule Engine performs CNN-based ML for context-aware learning about image context information and builds a knowledge-base with predefined rules. Incremental learning of images that can be gotten continuously by webcam provide guarantees for continuous context learning. Thus, it is possible to make the knowledge-base update incrementally.

**Figure 6:**
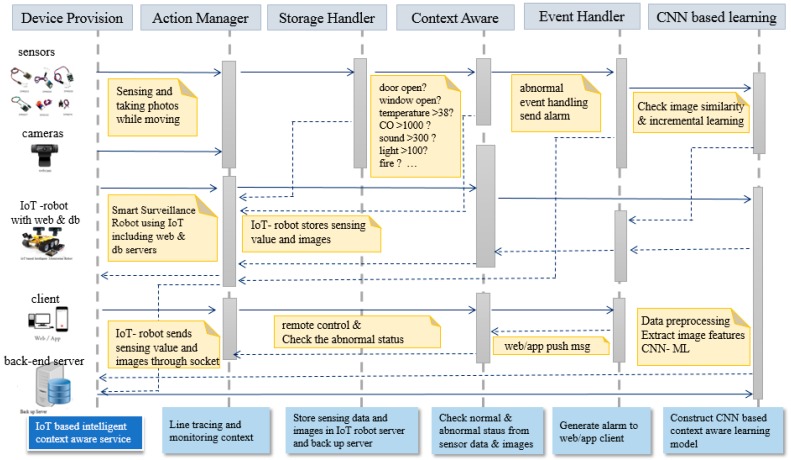
Sequence diagram of the IoT platform with an intelligent monitoring service.

Figure 6 shows the sequence diagram of the IoT platform with an intelligent monitoring system. Each situation is perceived according to the sensed values from sensors and a context-aware service is performed accordingly.

If the room temperature is out of the range of the predetermined value, an alarm is generated. Also, if the concentration of carbon monoxide is high, an alarm indicating that the air condition is improper is generated. When providing a surveillance and context awareness service according to images taken by the camera, an alarm is generated when a window or a door is opened.

**Figure 7:**
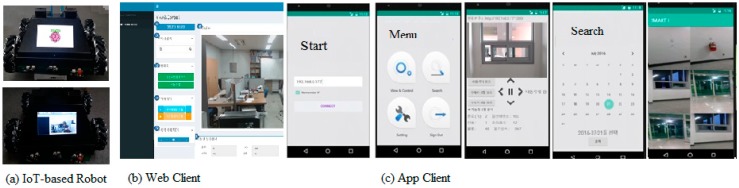
Graphical user interface of IoT platform with an intelligent monitoring robot. (**a**) IoT-based robot; (**b**) Web Client; (**c**) App Client.

It also shows the process of providing a continuous monitoring and context-aware service, according to whether the gas valve is locked or not, and whether or not there is a fire extinguisher at a predetermined position. The specification of the abnormal situation had been defined in advance. If the situation is abnormal, the context alarm information can be actively transmitted to the remote site. It can be utilized in user interfaces of various client environments by using standardized transmission technology-based on Wi-Fi network and TCP/IP protocol. We attached six sensors, a temperature sensor, sound sensor, light sensor, vibration sensor, carbon monoxide sensor, and flame detection sensor, to the robot in order to get the context of the specific environment. Figure 7 shows the implemented robot and graphical user interfaces. For the convenience of remote control and notification of abnormal alert app client was implemented as shown in Figure 7c. The ‘View & Control’ menu, from the main menu, allows the user to control the robot remotely in real time. The ‘Search’ menu can be used to search the images stored in specific date or time.

When the robot has performed monitoring, photos can be taken by its camera and compared with stored images of a normal state. When an abnormal status is detected, an alarm is sent to the manager via a smart phone. For user convenience, the app-client is able to control the robot remotely. In the case of image-context processing it is useful to apply convolutional neural network (CNN)-based machine learning, which is optimized for image recognition, and can be expected to give a higher performance in the accuracy of learning. A CNN-based context-aware learning model must be constructed in the back-end server. In next section, the design of the CNN-based learning model will be described.

## 4. CNN-Based Context-Aware Learning Model

In this section, we describe CNN-ML which is adopted for context-aware learning in this paper. First, we have designed the input layer, which defines the type and size of the image input function. The input size varies in accordance to different purposes. For classification tasks, the input size is typically the same size as the training images. However, for detection or recognition tasks, the CNN needs to analyze smaller parts of the image, so the input size must be at least the size of the smallest object in the data set. In this case, the CNN is used to process a (28 × 28)-RGB image. The middle layers, which are the core part of the CNN, consist of convolutional repetitive blocks, ReLU, and pooling layers. The convolutional layers are a set of filter weights which are updated during network training. The ReLU layer adds non-linear functions to the network. The pooling layers downsample data as they flow through the network. A deeper network can be created by repeating these basic layers.

Weights and activation functions are applied to the convolution output image of each channel. In the pooling layer, an output image with a size of 12 × 12 × 16 is generated by applying pulling with a size of 2 × 2 and a stride of 2 to an output image to which an activation function is applied. The pooling layer causes effective prevention of overfitting by reducing the size of the synthesized multi-layer output image so as to reduce the number of weights and the amount of computation. The image of the last pooling layer is transformed into a vector to be used as the input of the fully connected neural network. When the filter is applied, the edge information is lost, and the size of the output image is reduced.

In order to compensate for this, the size of the input image and the size of the output image can be made the same by performing a padding process.

**Figure 8:**
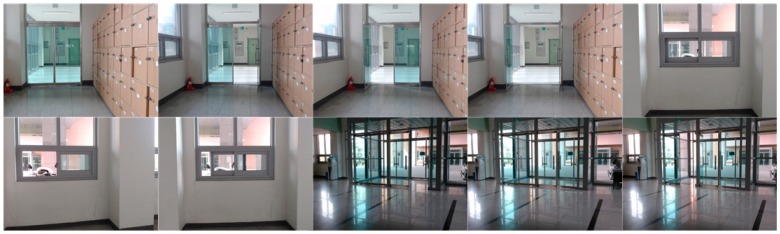
Images of opened/closed of door and window.

We evaluated the constructed CNN model which learns 3000 images taken by robots and classifies them into 10 situations. The scene captured by the robot stopped at a pre-set location inside the building is the state of opening/closing of the outer door, the inner door, and the window. It extracts ten images that distinguish between closed state and open state to be used as training data and test data. Figure 8 shows 10 types of images shot by the robot.

**Figure 9:**
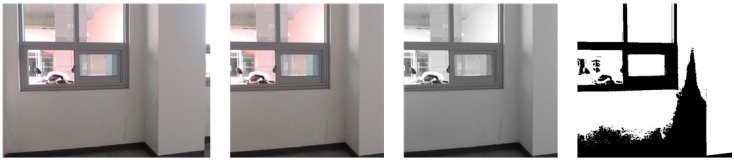
Convert process of original image. (**a**) Original image; (**b**) crop image; (**c**) gray-scale image; (**d**) binary image.

For the 640 × 480 color image taken at each designated location, the robot selects only the center and converts it to 480 × 480. After extracting the binary image through the grayscale image, it must be resized to 28 × 28 size and can be used as CNN input. The result of this process is shown as in Figure 9, which presents the original image in (a), the crop image in (b), the gray image in (c), and the binary image in (d).

The architecture of CNN model proposed in this paper is a variant of LeNet-5, and it consists of two convolution layers, two pooling layers, and two fully connected layers. It extracts features from the input image in a convolution layer which is generated by using a convolution of the input image and the weight. Weights are used as filters, and 3 × 3 filters or 5 × 5 filters are used for image learning in the proposed CNN model. The filter used in the first convolution layer is 5 × 5 × 32 and receives a 28 × 28 × 1 input and produces a 28 × 28 × 32 output. Next, as the maximum value pooling step, stride 2 is applied at the maximum value of the 2 × 2 window to generate the output of 14 × 14 × 32.

In the second convolution layer, a 14 × 14 × 32 input is used to generate a 14 × 14 × 64 output using a 5 × 5 × 64 filter, and as an input, a 7 × 7 × 64 output is generated in a second maximum value pooling. In the first fully connected layer, 7 × 7 × 64 (3136), which is the output of the second pooling layer, is changed to 1024 one-dimensional output. In the second fully connected layer, 1024 × 10 is generated, and then the final output layer is determined as 10 kinds of status using the softmax function. For example, a sigmoid function, a hyperbolic tangent function, or a ReLU (rectified linear unit) function can be used as an activation function in each convolution layer. Recently, the ReLU function has been used much more because of its high performance. We also use the ReLU activation function in this study. The ReLU function is simply defined as Equation (1).

*f*(*x*) = *max*(0, *x*) (1)

**Figure 10:**
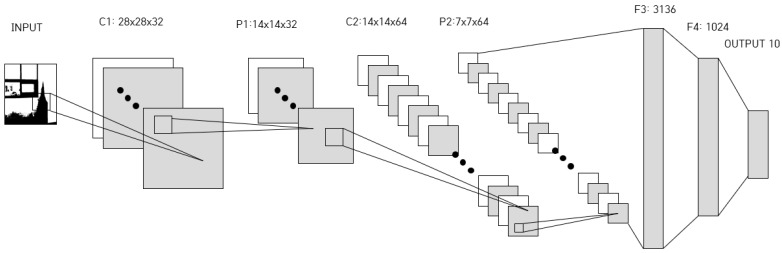
Architecture of extended CNN.

The ReLU function is a line with a slope of 1 if *x* > 0 and a slope equals 0 if *x* < 0. The architecture of extended CNN used in our study is shown in Figure 10.

## 5. Experimental Results and Discussion

The details of the extended CNN model, which consists of 2 convolution layers, 2 maximum pooling layers, and 2 full connection layers, is shown in Table 1. The active function uses ReLU and the input image is converted into a 28 × 28 size monochrome image.

**Figure 11:**
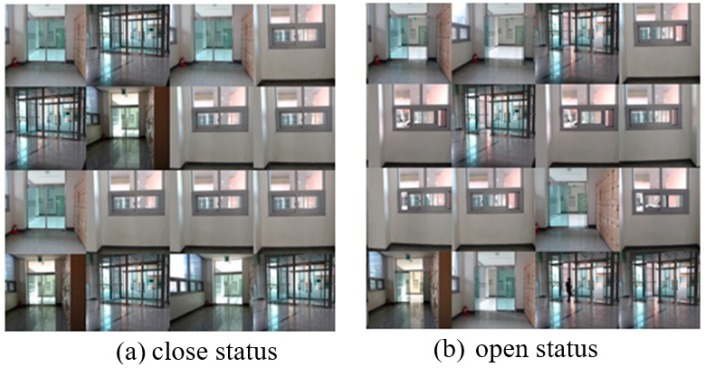
Images of windows and doors used in CNN Learning: (**a**) close status of doors and windows (**b**) open status of doors and windows.

We used 3000 images taken by the robots for the experiments. Among them 2700 images were used for learning data and 300 images were used for the test data. The learning process was performed through 10,000 epochs totally, and dropout was applied to reduce overfitting while learning. We adopted the cross-entropy function as a loss function and the ADAM for optimization algorithm. The learning rate is set at 0.05 and converts 1024 features into 10 classes (One-hot Encoding). The CPU used in the experiment is Intel i9-9900K (3.6 GHz) and the GPU is GeForce RTX2080Ti. Figure 11 shows the part of the data used in the learning and as shown in the figure status of the doors and the windows is closed or not.

Experimental results showed high recognition rate and it was demonstrated that the change of the dropout was not much influence on accuracy. For the comparison of optimization method, we demonstrated two optimization algorithms AdaGrad and ADAM and verified ADAM has better performance in learning accuracy than AdaGrad.

**Figure 12:**
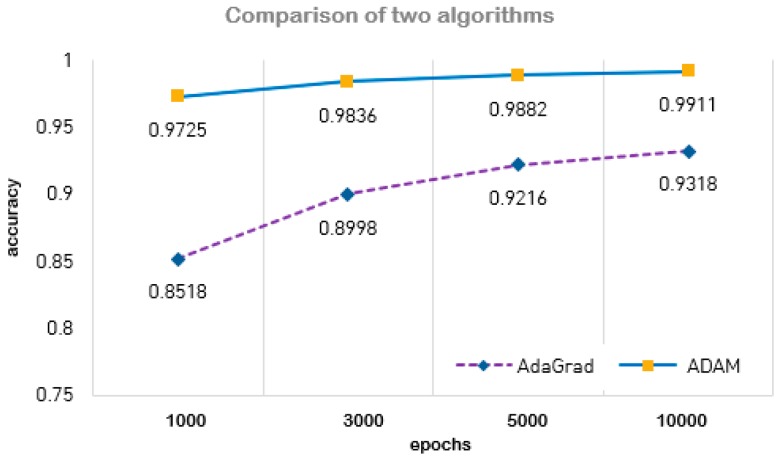
Accuracy comparison of two algorithms: ADAM and AdaGrad.

We tried to check accuracy according to epochs of 1000, 3000, 5000, and 10,000, and were able to verify the accuracy of both algorithms for each epoch. The accuracy rates of each algorithm are shown in Table 2 and Figure 12. In the case of ADAM, the accuracy was 0.9725 for 1000 epochs and 0.9911 for 10,000 epochs. Experimental results showed that 10,000 epochs using Adam optimization showed the highest performance. In experimental results of AdaGrad optimization, the accuracy was 0.8518 for 1000 epochs and 0.9318 for 10,000 epochs. As shown in Table 2, it was figured out that two algorithms had significantly different performance in learning accuracy.

**Figure 13:**
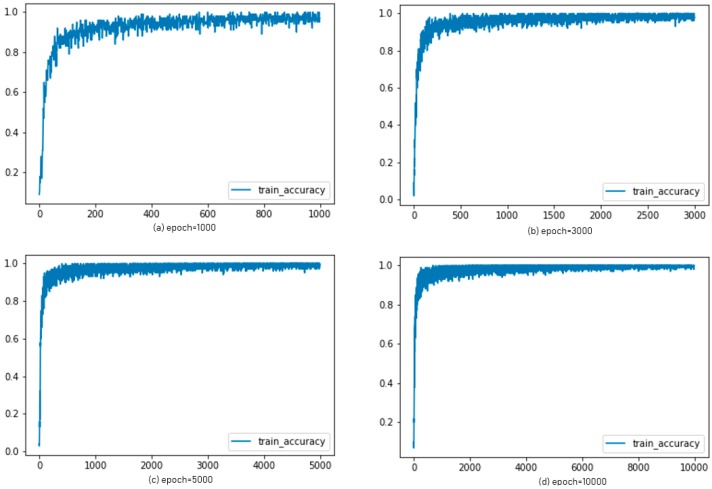
Experimental results: accuracy of ADAM for each epoch.

**Figure 14:**
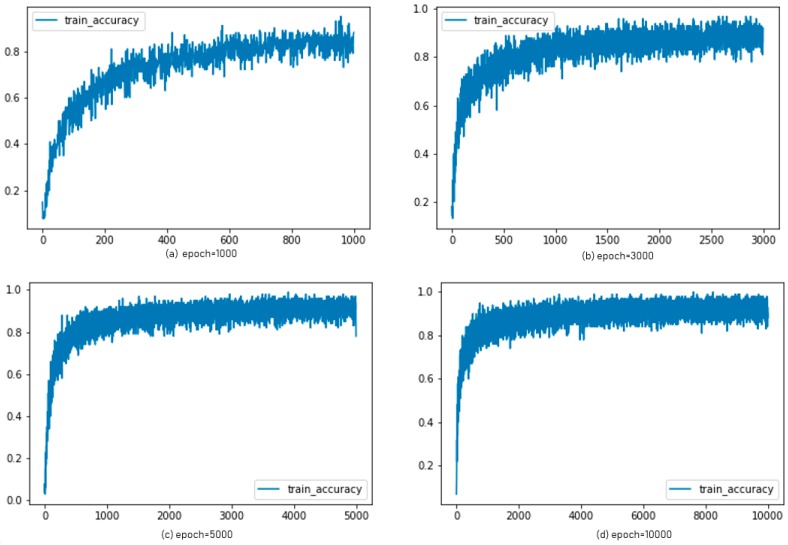
Experimental results: accuracy of AdaGrad for each epoch.

For the ADAM algorithm, the accuracy graphs were shown in Figure 13 with epochs of 1000, 3000, 5000, and 10,000, respectively. The graphs in Figure 14 showed the accuracy of AdaGrad according to each epoch.

## 6. Conclusions

IoT technologies bring innovations to wide intelligent robot services in the real-time applications domain. In this paper, we proposed an IoT platform with an intelligent monitoring robot, which can perform functions such as autonomous driving and situational awareness, real-time video transmission, and context-awareness of abnormal situations. We also designed the CNN model for an IoT-based intelligent monitoring robot to support enhancement of context-aware service.

The intelligent monitoring robot in the proposed IoT platform can be used as an active CCTV including an IoT server to overcome the problems of existing fixed CCTV. CNN-based ML is used to figure out whether the monitoring images were of normal or abnormal status in the case of image context. Servers were implemented in the proposed monitoring robot itself, which could perform real-time communication, processing sensing values, and shooting images from a webcam while monitoring. A CNN-based ML server was constructed in the back-end server for context-aware learning. We adopted the cross-entropy function as a loss function and the ADAM for optimization algorithm. The recognition rate was 0.9911 in experimental results.

The contribution of this paper is to improve the accuracy of context-aware learning for an IoT-based active-surveillance robot by applying CNN. The developed IoT-based monitoring robot can be used for rapid resolution of an abnormal situation of an image context in many areas such as the prevention and detection of intrusion, environment pollution, and potential disasters in a variety of fields. We are going to study to improve context-aware learning and to adapt it to actual situations such as in a factory, building, or home environment for practical use. However, the operation time of the robot was only about 5 h, and we exposed that there is a problem with the battery. In future work, we are going to study to improve the performance of the developed robot and to ensure battery efficiency.

## Figures and Tables

**Table 1:** Details of extended CNN model.

Layers	Parameter	Output Size
Input	-	28 × 28
Conv1	Filter Size: 5 × 5 Kernel: 32	28 × 28 × 32
Max Pool1	Filter Size: 5 × 5 Kernel: 2	14 × 14 × 32
Conv2	Filter Size: 5 × 5 Kernel: 64	14 × 14 × 64
Max Pool2	Filter Size: 5 × 5 Kernel: 2	7 × 7 × 64
FC1	Node: 3136	1024
FC2	Node: 1024	10

**Table 2:** Accuracy rate of two algorithm.

Optimization	Epochs	Accuracy
ADAM	1000	0.9725
	3000	0.9836
	5000	0.9882
	10,000	0.9911
AdaGrad	1000	0.8518
	3000	0.8998
	5000	0.9216
	10,000	0.9318
